# Supplementary material for: A multicolor suite for deciphering population coding of calcium and cAMP in vivo
Source: Nat Methods. 2024 Mar 21;21(5):897–907. doi: 10.1038/s41592-024-02222-9 (PMC11093745; doi:10.1038/s41592-024-02222-9)
Supplement: Supplementary file 1 — Supplementary Figs. 1–3 and Supplementary Tables 1 and 2. [file 41592_2024_2222_MOESM1_ESM.pdf]

nature methods

Article

https://doi.org/10.1038/s41592-024-02222-9

A multicolor suite for deciphering population coding of calcium and cAMP in vivo

In the format provided by the authors and unedited

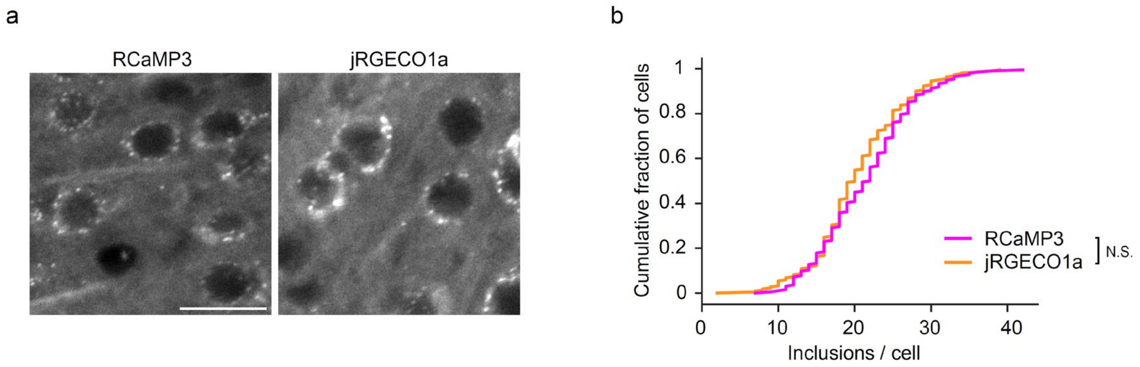

Supplementary Fig 1 | Inclusion analysis of RCaMP3

a, Representative confocal images of fixed tissue sections from the V1 after four weeks of jRGECO1a and RCaMP3 expression by AAV. Scale bar, 20 μm. **b**, Cumulative plot of inclusions per cell of jRGECO1a (orange) and RCaMP3 (magenta) expressed in L5 neurons. n = 221 cells in 2 mice (jRGECO1a), n = 196 cells in 2 mice (RCaMP3). Kolmogorov–Smirnov test. P = 8.3×10^{-2}.

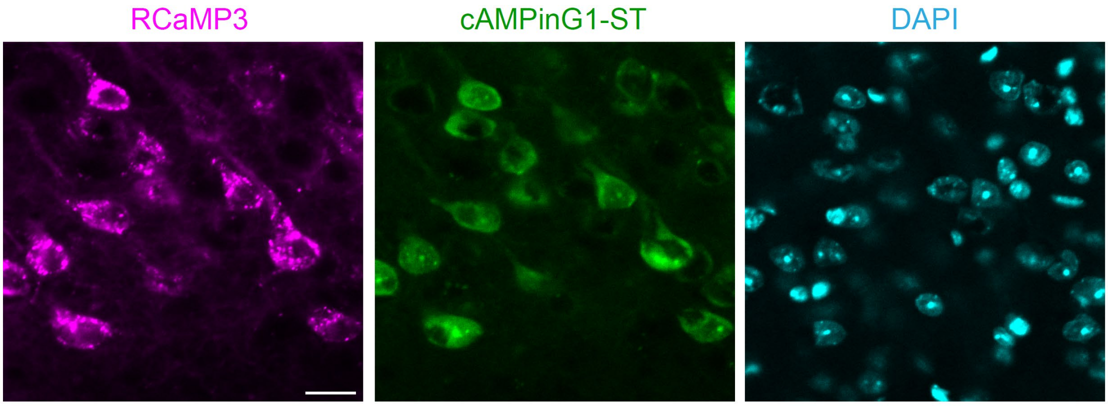

Supplementary Fig 2 | cAMPinG1-ST localization

Representative images of fixed tissue sections from L2/3 neurons in V1 expressing RCaMP3 and cAMPinG1-ST by AAV, counterstained with DAPI. Scale bar, 10 µm.

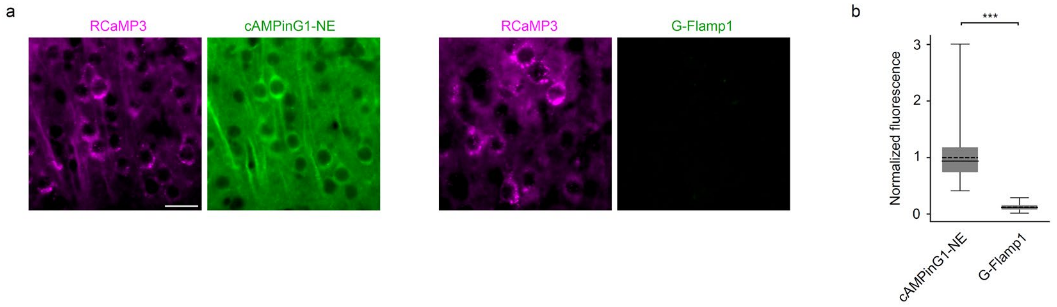

Supplementary Fig 3 | The fluorescent intensity of cAMP indicators

a, Representative confocal images of fixed tissue sections of RCaMP3 and cAMPinG1-NE (left) and RCaMP3 and G-Flamp1 (right) expressed in mouse V1 L2/3 neurons. RCaMP3 was used as an infection marker. Scale bar, 10 µm. **b**, Fluorescence intensities in confocal images of fixed tissue sections of cAMPinG1-NE and G-Flamp1 expressed in mouse V1 L2/3 neurons. n = 234 cells in 3 mice (jRGECO1a), n = 284 cells in 3 mice (RCaMP3). Unpaired two-tailed t-test. $P = 5.8 \times 10^{-144}$. Boxes indicate 25th and 75th percentiles, solid lines indicate median, dashed lines indicate mean, and whiskers indicate total range of data. Note that the expression efficacy of G-Flamp1 was lower than that of cAMPinG1-NE.

Supplementary Table 1. Comparison of cAMP sensors *in vitro*

Name	Type	This study		Literature values		Ref.
		ΔF/F	K_d (μM)	ΔF/F	K_d (μM)	
Flamindo2	Single-FP, Green	-0.80	2.5	-0.75	3.2	Odaka et al., PLoS One. 2014
R1α #7	FRET	-	-	0.38	0.037	Ohta et al., ACS Chem Biol. 2016
Pink Flamindo	Single-FP, Red	-	-	4.2	7.2	Harada et al., Sci Rep. 2017
R-FlincA	Single-FP, Red	-	-	6.0	0.30	Ohta et al., Sci Rep. 2018
cAMPFIRE-H	FRET	-	-	-	0.38	Massengill et al., Nat Methods. 2022
gCarvi	Single-FP, Green	2.2	6.5	1.5	2.0	Kawata et al., PNAS. 2022
G-Flamp1	Single-FP, Green	7.5	0.87	13.4	2.17	Wang et al., Nat Comm. 2022
G-Flamp2	Single-FP, Green	-	-	20.0	1.9	Liu et al., Front Pharmacol. 2022
cAMPinG1	Single-FP, Green	10.6	0.18	-	-	This study

-, not measured or described.

Supplementary Table 2. The pKa value of cAMP sensors

Name	pKa	
	cAMP-saturated	cAMP-free
Flamindo2	8.2	7.6
gCarvi	8.2	8.5
G-Flamp1	6.9	7.9
cAMPinG1	7.2	6.5
cAMPinG1mut	-	7.0
