# Supplementary material for: A multicolor suite for deciphering population coding of calcium and cAMP in vivo
Source: Nat Methods. 2024 Mar 21;21(5):897–907. doi: 10.1038/s41592-024-02222-9 (PMC11093745; doi:10.1038/s41592-024-02222-9)
Supplement: Supplementary file 2 — Reporting Summary [file 41592_2024_2222_MOESM2_ESM.pdf]

nature portfolio

Corresponding author(s): Tatsushi Yokoyama, Masayuki Sakamoto

Last updated by author(s): Jan 21, 2024

Reporting Summary

Nature Portfolio wishes to improve the reproducibility of the work that we publish. This form provides structure for consistency and transparency in reporting. For further information on Nature Portfolio policies, see our Editorial Policies and the Editorial Policy Checklist.

Statistics

For all statistical analyses, confirm that the following items are present in the figure legend, table legend, main text, or Methods section.

n/a	Confirmed	
☐	☒	The exact sample size (*n*) for each experimental group/condition, given as a discrete number and unit of measurement
☐	☒	A statement on whether measurements were taken from distinct samples or whether the same sample was measured repeatedly
☐	☒	The statistical test(s) used AND whether they are one- or two-sided<br>*Only common tests should be described solely by name; describe more complex techniques in the Methods section.*
☒	☐	A description of all covariates tested
☒	☐	A description of any assumptions or corrections, such as tests of normality and adjustment for multiple comparisons
☐	☒	A full description of the statistical parameters including central tendency (e.g. means) or other basic estimates (e.g. regression coefficient) AND variation (e.g. standard deviation) or associated estimates of uncertainty (e.g. confidence intervals)
☐	☒	For null hypothesis testing, the test statistic (e.g. *F*, *t*, *r*) with confidence intervals, effect sizes, degrees of freedom and *P* value noted<br>*Give P values as exact values whenever suitable.*
☒	☐	For Bayesian analysis, information on the choice of priors and Markov chain Monte Carlo settings
☒	☐	For hierarchical and complex designs, identification of the appropriate level for tests and full reporting of outcomes
☐	☒	Estimates of effect sizes (e.g. Cohen's *d*, Pearson's *r*), indicating how they were calculated

Our web collection on statistics for biologists contains articles on many of the points above.

Software and code

Policy information about availability of computer code

Data collection	Spark control software(v2.3) (TECAN), ZEN for SLM-880 (Carl-Zeiss), FV31S-SW for FVMPE-RS (Olympus), HC image (Hamatsu Photonics), pCLAMP (Molecular Devices), MCS for MOM (Sutter), Falcon for FASHIO-2PM (Nikon), LabVIEW (2021) (National Instruments), Neuroscience Studio (Doric)
Data analysis	ImageJ (Fiji 1.48), Python 3.8, Excel (Microsoft), Suite2p (https://github.com/MouseLand/suite2p), Cellpose (https://github.com/MouseLand/cellpose)

For manuscripts utilizing custom algorithms or software that are central to the research but not yet described in published literature, software must be made available to editors and reviewers. We strongly encourage code deposition in a community repository (e.g. GitHub). See the Nature Portfolio guidelines for submitting code & software for further information.

Data

Policy information about availability of data

All manuscripts must include a data availability statement. This statement should provide the following information, where applicable:

- Accession codes, unique identifiers, or web links for publicly available datasets
- A description of any restrictions on data availability
- For clinical datasets or third party data, please ensure that the statement adheres to our policy

cAMPinG1 and RCaMP3 plasmids and corresponding sequences were deposited to the RIKEN Bio-Resource Center (https://dna.brc.riken.jp/en/) for distribution with

the following catalog numbers: pN1-cAMPinG1 (RDB19691), pAAV-eSyn-cAMPinG1-NE-WPRE (RDB19692), pAAV-eSyn-cAMPinG1-ST-WPRE (RDB19693), pAAV-eSyn-RCaMP3-WPRE (RDB19694). The cAMPinG1 and RCaMP3 sequences are available from GenBank (accession number: LC728270 (cAMPinG1), and LC728271 (RCaMP3)).

Human research participants

Policy information about studies involving human research participants and Sex and Gender in Research.

Reporting on sex and gender	N/A
Population characteristics	N/A
Recruitment	N/A
Ethics oversight	N/A

Note that full information on the approval of the study protocol must also be provided in the manuscript.

Field-specific reporting

Please select the one below that is the best fit for your research. If you are not sure, read the appropriate sections before making your selection.

☒ Life sciences ☐ Behavioural & social sciences ☐ Ecological, evolutionary & environmental sciences

For a reference copy of the document with all sections, see nature.com/documents/nr-reporting-summary-flat.pdf

Life sciences study design

All studies must disclose on these points even when the disclosure is negative.

Sample size	Sample size was estimated based on prior experience and previous studies in the field. Sample sizes are reported for each experiment in the manuscript.
Data exclusions	For two-photon calcium and cAMP imaging, only cells recognized by Suite2p using calcium signals were included for further analysis.
Replication	We conducted the same experiments multiple times at least two times in different transfections (in vitro) or animals (in vivo). All experiments to replication were successful.
Randomization	Cells and animals were randomly allocated into experimental groups.
Blinding	Blinding was not performed in this study. The experimental condition were obvious to researchers and the analysis were performed objectively without human bias.

Reporting for specific materials, systems and methods

We require information from authors about some types of materials, experimental systems and methods used in many studies. Here, indicate whether each material, system or method listed is relevant to your study. If you are not sure if a list item applies to your research, read the appropriate section before selecting a response.

Materials & experimental systems

n/a	Involved in the study	
☒	☐	Antibodies
☐	☒	Eukaryotic cell lines
☒	☐	Palaeontology and archaeology
☐	☒	Animals and other organisms
☒	☐	Clinical data
☒	☐	Dual use research of concern

Methods

n/a	Involved in the study	
☒	☐	ChIP-seq
☒	☐	Flow cytometry
☒	☐	MRI-based neuroimaging

Eukaryotic cell lines

Policy information about cell lines and Sex and Gender in Research

Cell line source(s)	HEK293T cells were obtained from the American Type Culture Collection (CRL-11268).
Authentication	The cells were authenticated based on the morphology under microscope and growth rate.
Mycoplasma contamination	The cell lines were not tested for mycoplasma contamination.
Commonly misidentified lines (See ICLAC register)	No misidentified cell lines were used in this study.

Animals and other research organisms

Policy information about studies involving animals; ARRIVE guidelines recommended for reporting animal research, and Sex and Gender in Research

Laboratory animals	C57BL/6 and ICR wild-type animals used in this study were purchased from Japan SLC and experiments were performed using both male and female sex between 0–20 weeks of age. A2A-Cre mice ((B6.FVB (Cg)-Tg (Adora2a-cre) KG139Gsat/Mmucd)) were obtained from MMRRC and experiments were performed using male sex between 8 – 10 weeks of age. Mice were group-housed and kept on a 12-h light/dark cycle with ad libitum food and water at room temperature. The housing condition for were controlled at room temperature of approximately 22–24°C and a relative humidity of 40–60%.
Wild animals	No wild animals were used in this study.
Reporting on sex	Male and female animals were used.
Field-collected samples	No filed-collected samples were used in this study.
Ethics oversight	All experimental procedures were performed using protocols that were approved by the Institutional Animal Care and Use Committee at Kyoto University (Lif-K23008), University of Yamanashi (A1-20), RIKEN (W2022-2-012), and The University of Tokyo (P18-118).

Note that full information on the approval of the study protocol must also be provided in the manuscript.
